# Tribal implementation of a patient-centred medical home model in Alaska accompanied by decreased hospital use

**DOI:** 10.3402/ijch.v72i0.20960

**Published:** 2013-08-05

**Authors:** Janet M. Johnston, Julia J. Smith, Vanessa Y. Hiratsuka, Denise A. Dillard, Quenna N. Szafran, David L. Driscoll

**Affiliations:** 1 Institute for Circumpolar Health Studies, University of Alaska Anchorage, Anchorage, AK, USA; 2 Southcentral Foundation, Anchorage, AK, USA

**Keywords:** Patient-Centered Medical Home, Alaska Native, asthma, inpatient hospitalization, time series analysis

## Abstract

**Background:**

Between 1995 and 1998, tribally owned Southcentral Foundation (SCF) incrementally assumed responsibility from the Indian Health Service (IHS) for primary care services on the Alaska Native Medical Center (ANMC) campus in Anchorage, Alaska. In 1999, SCF began implementing components of a Patient-Centered Medical Home (PCMH) model to improve access and continuity of care.

**Objective:**

To evaluate hospitalisation trends before, during and after PCMH implementation.

**Design:**

Time series analysis of aggregated medical record data.

**Methods:**

Regression analysis with correlated errors was used to estimate trends over time for the percent of customer-owners hospitalised overall and for specific conditions during 4 time periods (March 1996–July 1999: SCF assumes responsibility for primary care; August 1999–July 2000: PCMH implementation starts; August 2000–April 2005: early post-PCMH implementation; May 2005–December 2009: later post-PCMH implementation). Analysis was restricted to individuals residing in Southcentral Alaska and receiving health care at ANMC.

**Results:**

The percent of SCF customer-owners hospitalised per month for any reason was steady before and during PCMH implementation, declined steadily immediately following implementation and subsequently stabilised. The percent hospitalised per month for unintentional injury or poisoning also declined during and after the PCMH implementation. Among adult asthma patients, the percent hospitalised annually for asthma declined prior to and during implementation and remained lower thereafter. The percent of heart failure patients hospitalised annually for heart failure remained relatively constant throughout the study period while the percent of hypertension patients hospitalised for hypertension shifted higher between 1999 and 2002 compared to earlier and later years.

**Conclusion:**

Implementation of PCMH at SCF was accompanied by decreases in the percent of customer-owners hospitalised monthly for any reason and for unintentional injury and in the percent of asthma patients hospitalised annually for asthma. Increased accessibility to empanelled care teams may have contributed to decreased need for hospitalisation.

The tribally owned and operated Southcentral Foundation (SCF) provides health care to Alaska Native and American Indian (AN/AI) people in Southcentral Alaska. Between 1995 and 1998, SCF incrementally assumed responsibility from the Indian Health Service (IHS) for primary care services on the Alaska Native Medical Center (ANMC) campus in Anchorage, Alaska. In January 1999, SCF became co-owner of ANMC, along with the Alaska Native Tribal Health Consortium. In August 1999, SCF began to implement a new model of care that included components of the Patient-Centered Medical Home (PCMH). This new model, referred to as the Nuka model, focuses on relationships with the aim of improving access and continuity of care (1). SCF now refers to their patients as customer-owners because they are both consumers of health care and, as tribal members, also owners of the health care system.

According to the Agency for Healthcare Research and Quality (AHRQ), the medical home model provides the possibility to improve health care by transforming how primary care is organised and delivered. Building on the work of a large community of physicians, researchers and policymakers, AHRQ defines a medical home as a model of the organisation of primary care that delivers the core functions of primary health care (2). The medical home encompasses 5 functions and attributes:

- Comprehensive care
- Patient-centred
- Coordinated care
- Accessible services
- Quality and safety

Major features of the Nuka model include

- Empanelment with patients matched by self-selection or assignment to an integrated care team (ICT).
- Open access providing increased access to services through open scheduling, expanded office hours and increased availability of electronic communication between patients and ICT members.
- Team-based care whereby care is delivered by multidisciplinary teams rather than by an individual provider.

In 2010, SCF attained Level 3 Patient-Centered Medical Home™ recognition status by the National Committee for Quality Assurance for the Nuka model.

The purpose of this report is to describe changes in health care utilisation that occurred before, during and after the SCF PCMH implementation. Specifically, we report on changes in the overall use of inpatient services as well as in hospitalisations for certain chronic conditions sensitive to ambulatory care. We also look in more detail at the effects on adult asthma care because adult asthma has been identified by AHRQ as an ambulatory care-sensitive condition, meaning that good outpatient care can potentially prevent the need for hospitalisation (3), and because diseases of the respiratory system are the second leading cause of hospitalisation in the Alaska Tribal Health System (4).

## Methods

The results reported here are from our previously described mixed methods study to assess the impact of a PCMH model of care on the nature and quality of health care delivery at the SCF in Anchorage, Alaska (5). The study included quantitative data analysis of aggregate medical record data and qualitative analysis of primary data collected through semi-structured, individual interviews with patients, health care providers, other employees and tribal leaders. This report is based on time series analysis of the quantitative data, focusing on changes in hospitalisations before, during and after initiation of the PCMH implementation. We also delve into more detail related to adult asthma, reporting on changes in percent of adult customer-owners with any visit for asthma. We have previously reported on changes in Emergency Care (EC) use overall and separately for adult asthma and unintentional injury (5).

In order to evaluate changes in health care utilisation related to the PCMH implementation that started in 1999, we analysed monthly and annual data starting in 1996, the earliest year for which complete data were available. Rates were calculated using monthly and yearly event counts and patient counts extracted from the Resource and Patient Management System (RPMS), SCF's electronic medical record system. Data were only available as counts at the group level with no individual level data provided.

### Study population

The study population included those individuals residing in the Anchorage area who sought care at the ANMC or at SCF clinics on the ANMC campus during the study period – March 1996 through December 2009. ANMC and SCF provide services to all IHS beneficiaries, including AN/AI people who live in the Anchorage area and who receive primary care services from SCF and those who live in other areas of the state who may be transported to ANMC for tertiary care. A small number of non-IHS beneficiaries, such as Commissioned Corp officers and local residents in need of time critical emergency treatment, also receive care at ANMC.

In order to limit the analysis to patients who receive primary care from SCF, the sampling population was limited to individuals with a city of residence in the Anchorage area who sought care at least once on the ANMC campus during the 3 years prior to the time period for which data was being extracted. For example, data extracted for March 2002 is based on all patients with a visit to the ANMC campus from March 1999 to February 2002. Annual data for 2003 is based on patients with a visit to the ANMC campus in 2000, 2001 or 2002. Visits coded as pharmacy, telephone or administrative were excluded. RPMS retains the most recent city of residence, therefore residence was based on the city of residence at the time of data extraction.

### Ethics review

The IHS Alaska Area and University of Alaska Anchorage Institutional Review Boards, and tribal leadership of SCF and the Alaska Native Tribal Health Consortium approved all study protocols.

### Outcome measures

For this report, we analysed data on hospitalisations for:

- Any reason among all customer-owners
- Unintentional injury, such as from a car accident or fall, or accidental poisoning among all customer-owners
- Asthma among adult asthma patients
- Heart failure among adult heart failure patients
- Hypertension among adult hypertension patients
- Chronic liver disease among all adult customer-owners

For asthma, we also analysed monthly percentages of all adult customer-owners with any medical encounter for asthma – including clinic visits, EC visits and hospitalisations

Asthma was defined using ICD-9 codes 493.xx, excluding customer-owners with certain previous ICD-9 codes indicating cystic fibrosis, lung anomalies, perinatal chronic respiratory disease and related conditions. Heart failure was defined as ICD-9 codes 428.xx. Hypertension was defined as ICD-9 codes 401.xx–404.xx, excluding those with cardiac procedure or kidney disease ICD-9 codes. Chronic liver disease was defined as ICD-9 codes 571.xx. Unintentional injury or poisoning was defined using ICD-9 injury and poisoning codes (800–995.7x) excluding complications of medical and surgical care, self-inflicted injury or poisoning and intentional abuse.

### Analytic methods

As described previously, levels and trends were estimated using segmented regression analysis with autocorrelation (5). Candidate variables for the regression models included a monthly counter for the entire study period (March 1996–December 2009) to estimate the baseline study-wide trend as well as monthly counters for each of 3 discrete later study segments to estimate changes from the baseline trend (Segment 1: August 1999–July 2000 [PCMH implementation starts]; Segment 2: August 2000–April 2005 [Early post-PCMH implementation]; Segment 3: May 2005–December 2009 [Later post-PCMH implementation]) (6). Candidate variables also included dichotomous variables to estimate level changes for each of the study segments. Final models were fit based on the significance of the candidate variables in the multivariate models, the Akaike Information Criterion (AIC) and diagnostics including plots of residuals and white noise probabilities.

**Fig. 1:**
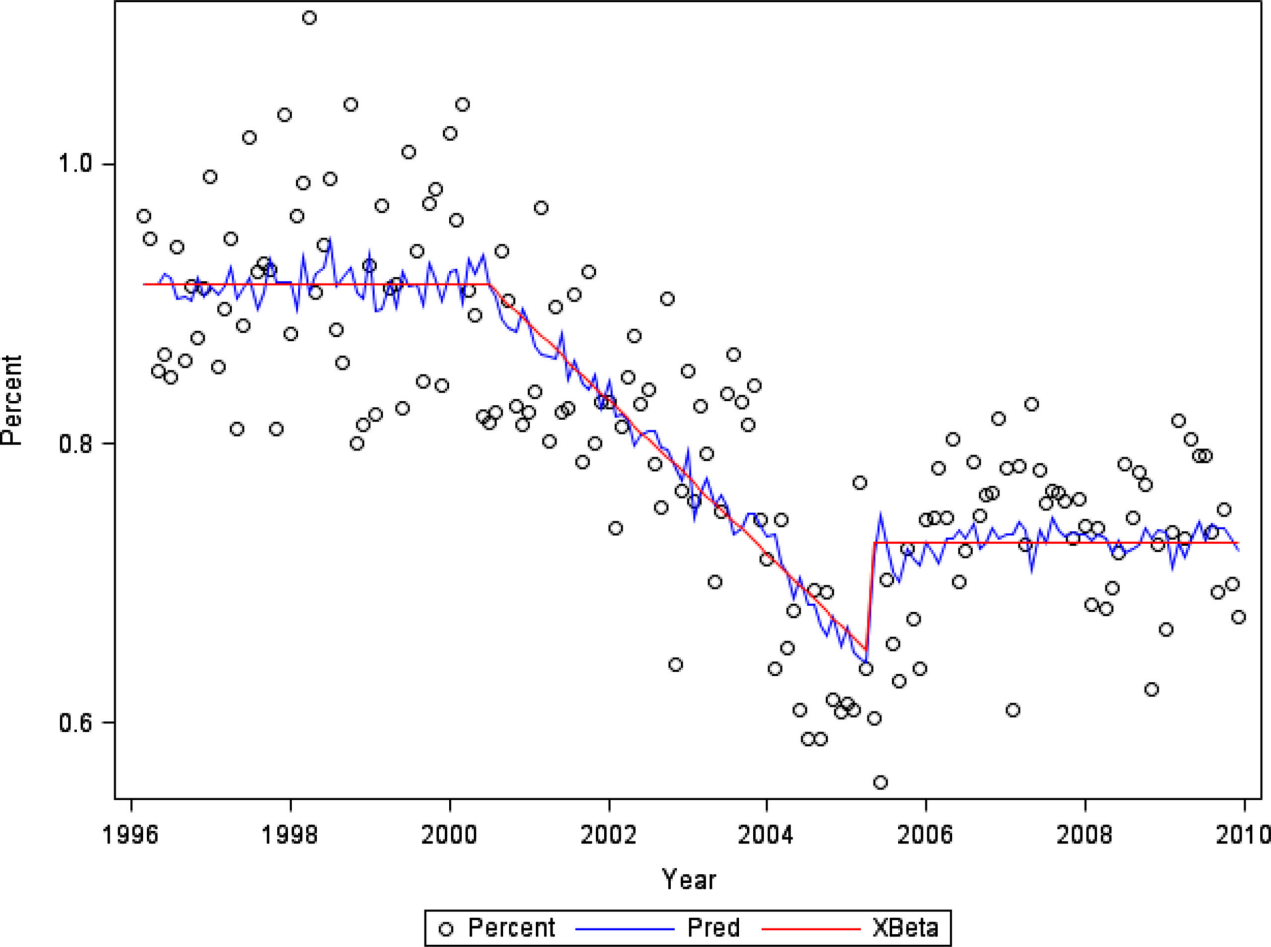
Percent of customer-owners with one or more hospitalisations per month for any reason.

Regression analyses were conducted using SAS software using PROC AUTOREG (7). Figures 1 and 3 indicate the observed percentage (Percent) for each month; the series of structural predicted values (Xβ), accounting for estimated changes in trend and level; and the series of predicted values (Pred) from the full model, including both the structural part and the predicted values of the error process.

## Results

As shown in Table I, the SCF customer-owner population residing in the Anchorage area increased by 69% from 28,100 in 1996 to 47,464 in 2009. The mean number of SCF customer-owners with one or more EC visits in 1 month rose from 2,123 in 1996 to 2,772 in 2009 while the mean percentage of customer-owners with one or more EC visits in the month dropped from 7.6% in 1996 to 5.8% in 2009. Similarly, the mean number of SCF customer-owners with one or more hospital admissions in 1 month rose from 255 in 1996 to 352 in 2009 while the mean percentage of customer-owners with one or more hospital admissions in 1 month dropped from 0.9 to 0.7%.

**Table I:** Mean monthly population, patients with at least 1 emergency care visit and patients with at least 1 hospital admission (Anchorage area patients, 1996–2009)

	SCF patient population		EC visits		Hospital admissions	
			
Year	N	% increase from previous year	N	%	N	%
1996	28,100	N/A	2,123	7.56	255	0.91
1997	29,545	5.14	2,622	8.87	271	0.92
1998	31,768	7.52	2,941	9.26	295	0.93
1999*	33,650	5.92	2,051	6.10	307	0.91
2000	35,524	5.57	2,843	8.00	318	0.90
2001	37,610	5.87	2,764	7.35	320	0.85
2002	39,446	4.88	2,690	6.82	316	0.80
2003	41,101	4.20	2,851	6.94	329	0.80
2004	42,122	2.48	2,766	6.57	275	0.65
2005	42,928	1.91	2,737	6.38	280	0.65
2006	44,017	2.54	2,533	5.75	335	0.76
2007	45,259	2.82	2,599	5.74	341	0.75
2008	46,350	2.41	2,736	5.90	336	0.72
2009	47,464	2.40	2,772	5.84	352	0.74

* Some EC visits were coded for other clinics during 4 months in 1999, resulting in lower EC visit counts.

### Inpatient hospitalisations

The percentage of customer-owners with one or more hospitalisations in each calendar month fluctuated at a level around 0.91% prior to and during the PCMH implementation (Fig. 1). During the early post-PCMH implementation period, the percentage decreased by 0.005% per month before flattening out during the later post-PCMH implementation period to a level of approximately 0.73% (p<0.001 for model intercept and regression coefficients). This model has a regression R-square of 0.57 and a total R-square of 0.66.

**Fig. 2:**
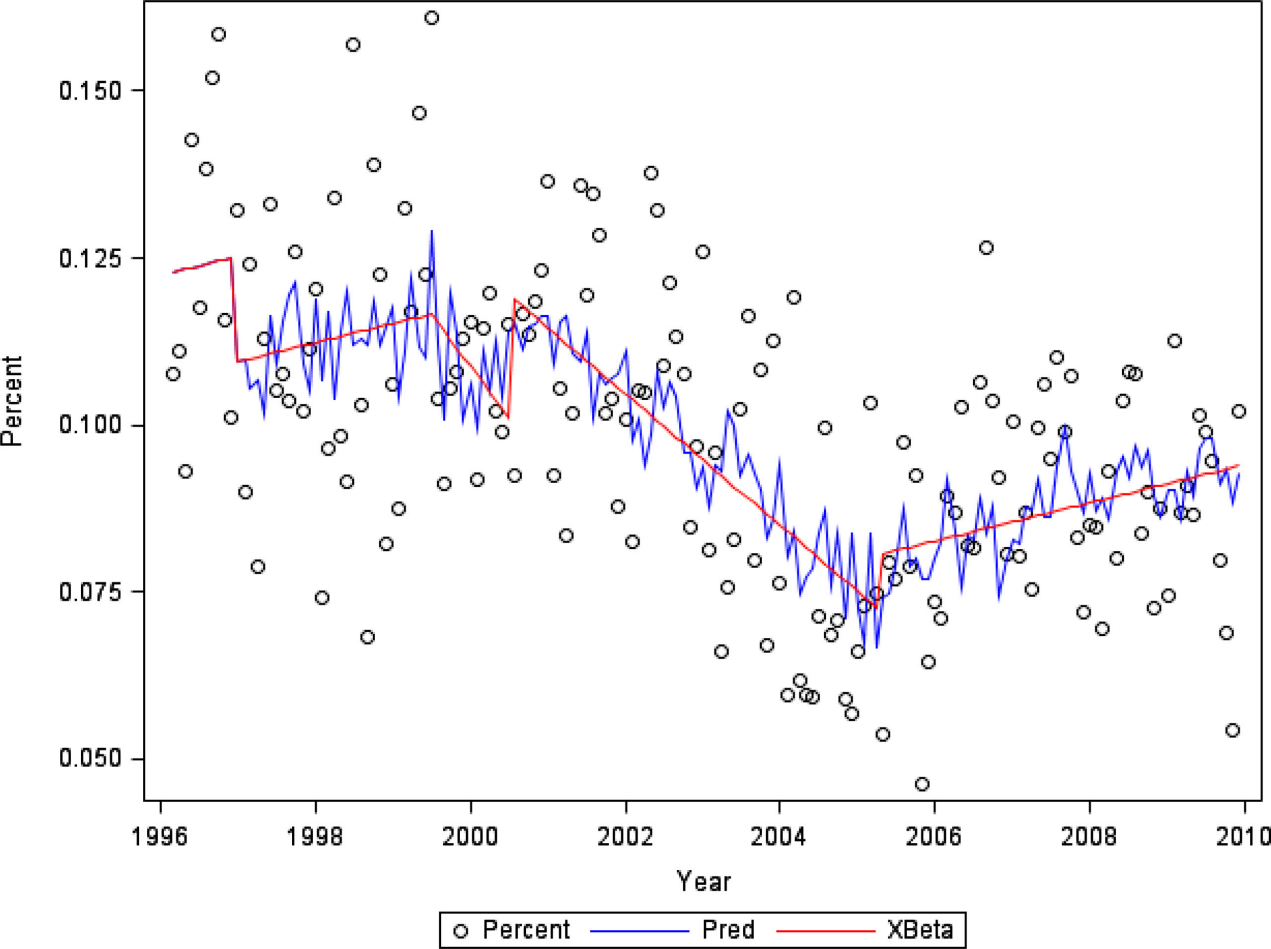
Percent of customer-owners with one or more hospitalisations per month for unintentional injury or poisoning.

The percentage of customer-owners with one or more hospitalisations for unintentional injury in each calendar month increases slowly prior to the PCMH implementation (β=0.0002%, p=0.067) and then decreased during both the first year of implementation (β=−0.0015%, p=0.035) and the early post-implementation period (β=−0.0011%, p<0.001). The later post-implementation saw a return to the slow rate of increase seen during the pre-implementation period (β=0.0002%, p=0.067; Fig. 2). This model has a regression R-square of 0.29 and a total R-square of 0.42.

### Asthma care

In order to better understand the effects on the PCMH implementation on chronic conditions considered sensitive to primary care, we examined changes over time in the percentage of customer-owners with asthma-related medical encounters. The percentage of customer-owners with any medical encounter involving an asthma diagnosis – including clinic visits, EC visits and hospitalisations – remained relatively stable with a mean level of approximately 0.4% prior to the PCMH implementation. During the initial implementation, the mean level rose to approximately 0.7% but then declined to approximately 0.6% during the early- and late post-PCMH implementation periods (p<0.001 for all). This model has a regression R-square of 0.45 and a total R-square of 0.53.

**Fig. 3:**
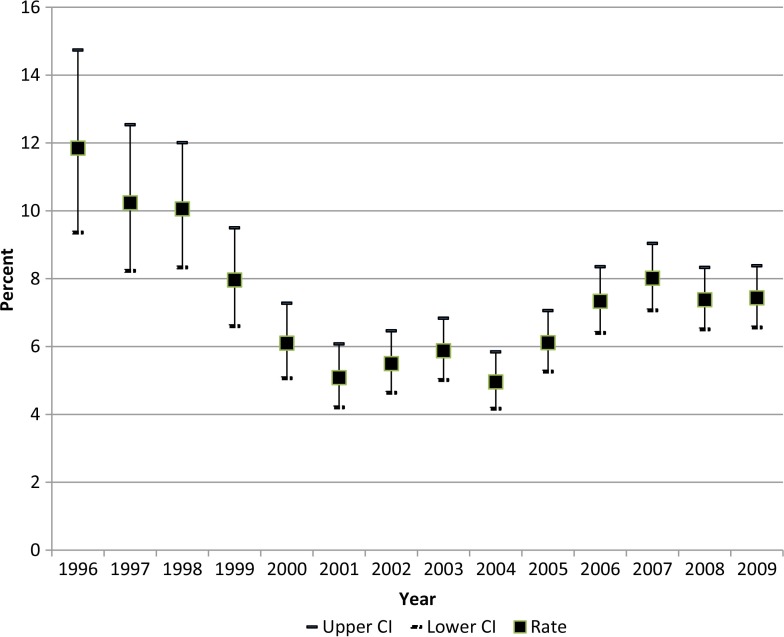
Percent of adult asthma patients with one or more hospitalisations per year.

The percentage of adult asthma patients hospitalised each year declined from a high of 14.7% in 1996 to 6.1% in 2001 (Fig. 3). This percentage remained under 7.0% through 2004 and then stayed between 7.0 and 9.0% from 2005 to 2009.

### Hospitalisations for other chronic conditions

In order to further evaluate the effect of the PCMH implementation on hospital use, hospitalisation data for 3 other chronic conditions sensitive to ambulatory care – heart failure, hypertension and chronic liver disease – were also extracted and analysed. While there were insufficient numbers of events to analyse hospitalisations for these conditions using monthly data, some trends emerged based on purely visual examination of graphical representations of the point estimates and 95% confidence intervals (CI) for the percentage of patients hospitalised annually for these conditions. Specifically, the percent of adult heart failure patients hospitalised for heart failure remained relatively constant for the majority of the study period from 1996 to 2009. The percent of adult hypertension patients hospitalised for hypertension exhibited level shifts with percentages near 15% during 1996 and 1997, rising closer to 20% for 1998 through 2002 and then settling back below 15% from 2004 to 2009. The percent of all customer-owners hospitalised for chronic liver disease showed a sine wave pattern with a low of 0.26% in 1997, rising to a high of 0.40% in 2001, then falling back to 0.22% in 2004 and once again rising to 0.34% in 2008. (Data available on request)

## Discussion

Inpatient hospitalisations among all patients for any reason were steady before and during initial PCMH implementation, declined steadily immediately following implementation and subsequently stabilised. Hospitalisations for unintentional injury or poisoning also declined during and after the PCMH implementation; however, they showed a slight upward trend before the implementation and then again in the later post-implementation phase of the study.

The percentage of adult SCF customer-owners with one or more medical encounters for asthma was level before the PCMH implementation, then increased during the implementation and remained steady, at a significantly higher level, after the implementation through the end of the study period. Among adult asthma patients, however, the percentage with one or more hospitalisations per year declined prior to and during the implementation and remained lower after the implementation. As reported previously, the percentage of adult asthma patients with one or more EC visits during each 2-month period also declined prior to, during and immediately after the PCMH implementation, with a slight rise in the later post-implementation phase (5).

Major features of the SCF PCMH implementation included empanelment of patients to an ICT; increased access to services and ICT members; and multidisciplinary team-based care. Improved access to outpatient care from a team of providers who are able to address medical concerns earlier before they become serious or life threatening may be 1 reason for reductions shown here in the percent of SCF customer-owners in need of hospital-based care. The asthma findings – with increases in the percentage of adult asthma patients seeking any care for asthma accompanied by decreases in the percentage of adult asthma patients requiring hospitalisation – also support this hypothesis.

To date, there are few published peer-reviewed evaluations of the PCMH model using health outcomes such as hospitalisations. This may not be surprising, given that in 2010 a majority of PCMH pilot projects did not have well-developed implementation plans (8). However, a 2009 national study of 7 health plans in 5 states did find that the more a practice implemented medical home concepts, the lower the rates of hospitalisation for children with chronic conditions in that practice (9).

We hypothesised that hospital use among all SCF customer-owners for unintentional injury or poisoning would be less affected by PCMH implementation than hospital use for all SCF customer-owners for any cause; however, we found a similar decrease in both measures following the implementation. The decrease in hospitalisation for unintentional injury may be related to a decrease in serious injuries. According to the Alaska Native Epidemiology Center, there was a 17% decrease from 1999 to 2005 in unintentional injury deaths among Alaska Native people. They also reported a decrease in motor vehicle related injury hospitalisations from 1999 to 2003 (10).

### Limitations

It is impossible to determine causality from time series data alone. The purpose of this report is to describe changes in health care utilisation before, during and after the PCMH implementation. Additional manuscripts that include findings from the qualitative data will shed more light on the various features of the implementation that may be related to the changes reported here.

These analyses are based on data that was originally collected for health care and administrative purposes, not for research. While we worked closely with the SCF Data Services department to ensure the accuracy of the data, it is possible that some errors still remain. Also, because only group data were available – event counts and patient counts – we were not able to adjust for changes in age and sex distributions during the course of the study.

IHS beneficiaries with additional health insurance, either private or government provided such as Medicare or Medicaid, have choices available to them when seeking health care. With other hospitals and health care facilities in the Anchorage area, it is unknown how many IHS beneficiaries seek some or all of their health care outside of the ANMC campus and whether PCMH had an effect on rates of such use. Therefore, we are not able to estimate the effect that access to alternate health care facilities has on these results.

In addition to the PCMH implementation, there were many changes during the study period that may have affected hospitalisation rates and other use of health care services. These changes include transfer of state-wide tertiary care from IHS to SCF and the Alaska Native Tribal Health Consortium, a consortium of state-wide non-profit Alaska Native tribal health organisations, including SCF; evolving treatment practices for asthma and other health conditions (11); and continued movement of AN/AI people to and from Anchorage and other areas of the state due to economic, social services and health care reasons (12).

## Conclusions

Implementation of a PCMH model at SCF was accompanied by decreases in the percent of customer-owners hospitalised monthly for any reason and for unintentional injury, and in the percent of asthma patients hospitalised annually for asthma. Increased accessibility to empanelled care teams may have contributed to decreased need for hospitalisation.
